# Evolution of sagittal spinal shape for the development of thoracic ossification of ligamentum flavum

**DOI:** 10.1097/MD.0000000000036543

**Published:** 2023-12-08

**Authors:** Yong Hyuk Choi, Myung Hoon Shin, Jong Tae Kim

**Affiliations:** a Department of Neurosurgery, Incheon St Mary's Hospital, College of Medicine, The Catholic University of Korea.

**Keywords:** Roussouly classification, sagittal alignment, thoracic ossification of ligamentum flavum

## Abstract

Thoracic ossification of the ligamentum flavum (TOLF) is a rare pathology for which limited research exists. While it is known that mechanical factors play a role in the development of TOLF, little is currently understood about the sagittal alignment and related mechanical stress involved in its development. This study aims to describe the sagittal alignment of patients with TOLF based on the pathologic evolution of the Roussouly classification. The current study evaluated the preoperative Roussouly type in consecutive patients who underwent posterior decompressive laminectomy with or without posterior screw fixation for TOLF between January 2015 and December 2021. The post-evolution sagittal alignments were analyzed using the classic Roussouly classification based on sacral slope (SS). To determine the pre-evolution Roussouly type, the patients were retrospectively classified using their individual PI and PT values. Lumbopelvic parameters and morphological index including inflection point (IP), lumbar apex (LA), and lordosis distribution index (LDI) were also evaluated. Forty-three patients (21 women and 22 men) were included; their mean age was 64.21 ± 11.01 years (range 43–81). The most affected level was T10-11 (48.83%). The mean PI was 50.81 ± 9.56°, the mean SS was 33.11 ± 8.61°and the mean PT was 17.69 ± 7.89°. According to the post-evolution Roussouly classification, type 2 shape was the most frequently observed type (n = 23, 53.5%) in the post-evolution classification while type 3 was the most common type observed in the pre-evolution classification (n = 22, 51.5% and *P* = .00). The level of IP and LA in type 3 moved caudally (around L2 and L4/5 level, respectively) and the LDI increased (77.98 ± 8.08%) than the normal standard value. The authors found that the majority of the patients had a false type 2 spine, which had evolved pathologically from Roussouly type 3 and exhibited increased LDI, a lowered level of IP, and a lowered level of LA. These changes of spinal shape, including the transition to long hypolordosis and increased length of the thoracic kyphosis, may have accentuated tensile stress at the lower thoracic spine and contributed to the development of TOLF.

## 1. Introduction

Thoracic spinal stenosis (TSS) is a rare group of clinical syndromes that result from the narrowing of the spinal canal and compression of the thoracic spinal cord. The pathological factors that contribute to TSS include degenerative ligament hypertrophy and ossification, disc herniation, and osteophytes around the edges of the vertebral body and facet joint.^[1]^ Although thoracic myelopathy caused by TSS is uncommon, the clinical outcome is generally poor. This is due to several factors, including the shaky blood supply to the thoracic spinal cord, the kyphotic alignment of the thoracic spine which limits the ability of the cord to shift backward in response to ventral compression, and the slow-progressing nature of the condition which can result in delayed diagnosis and irreversible neurological damage.^[2–4]^

The 3 main pathologies contributing to TSS’s progression are thoracic ossification of the ligamentum flavum (TOFL), thoracic disc herniation, and ossification of the posterior longitudinal ligament. Among these, thoracic ossification of ligament flavum (TOLF) is the most common cause of TSS.^[1]^ It is characterized by the replacement of the ligamentum flavum with mature bone tissue containing many chondrocytes or chondroblast-like cells in the stroma of the degenerated ligament.^[5]^ Given the selectively high prevalence in eastern Asian countries,^[6,7]^ researchers have sought to understand the genetic factors that may contribute to its pathogenesis. However, the explicit mechanism behind TOLF remains unclear, although various authors have endorsed the significance of biological factors in its development.^[8,9]^ The local factor of mechanical stress is also thought to play a significant role in the pathogenesis of TOLF. It has been commonly reported that the most frequently affected segment is the lower thoracic spine,^[1,6,10,11]^ where the high tensile force in the posterior column can easily lead to ligament degeneration and the transformation of elastic fibers into ossified tissue.^[5]^

The amount of mechanical stress around the thoracic spine depends on various factors, including the shape, alignment, and degeneration of the vertebral column. As the patient-specific pelvic incidence (PI) determines the shape of lumbar lordosis (LL) via sacral slope (SS) and the reciprocal thoracic kyphosis (TK) would be made, the authors hypothesized that the TOLF might prefer to evolve from a certain alignment shape. In this study, we delineated the sagittal alignment of a patient post-decompressive surgery for TOLF, utilizing the Roussouly classification system.^[12]^

Additionally, we tried to offer a plausible elucidation for the evolution of normal Roussouly-type shapes into their pathological counterparts.

## 2. Methods

The authors conducted a retrospective analysis of medical records from their institution, with approval from the institutional review board. The study included consecutive patients who underwent posterior decompressive laminectomy with or without posterior screw fixation for TOLF between January 2015 and December 2021. Patients who had previously undergone thoracic or lumbar surgery or had a history of spondylitis, trauma, tumors, degenerative scoliosis with a lumbar Cobb angle greater than 15 degrees, or congenital deformities were excluded. The laminectomy was confined to the compressed spinal cord segment, and the TOLF was completely resected or floated in all patients. Additional stabilization using screw fixation was performed in patients who required extensive decompression of more than 50% of the bilateral facetectomy or demonstrated segmental instability preoperatively.

The clinical information from the electronic medical record was reviewed for demographics, including age, gender, height, body weight, and body mass index (BMI). In addition, the distribution of TOLF was assessed based on the operation record where the decompressed segment of TOLF was documented.

Radiographic measurements were taken from a preoperative whole spine lateral radiograph, which included the external auditory canal and proximal femur. The collected radiographic parameters included the sagittal vertical axis (SVA), which is the distance from the C2 plumb line to the posterosuperior corner of the S1 upper endplate; the thoracic pelvic angle (TPA), which is the angle between 2 lines drawn from the bicoxofemoral axis to the center of the S1 endplate and the center of the T1 vertebral body; the TK, which is the Cobb angle between T4 and T12; the SS, which is the angle between the horizontal line and the line forming the S1 upper endplate; the pelvic tilt (PT), which is the angle between the line connecting the center of the S1 upper endplate to the bicoxofemoral axis and the vertical line; the PI, which is the angle between the line perpendicular to the line forming the S1 upper endplate at its center and the line connecting this point to the bicoxofemoral axis; and the LL, which is the Cobb angle between L1 and S1. Positive values are considered lordosis, and negative values are considered kyphosis.

Lordosis distribution index (LDI),^[13]^ defined as the magnitude of lower-arc lordosis relative to the total lordosis, was calculated using the following formula: L4-S1 lordosis/L1-S1 lordosis × 100. The inflection point (IP), defined as the point in the spine where TK becomes LL, was adopted to determine the demarcation at which the direction of the curve alters and the number or length of kyphotic or lordotic vertebrae.^[14]^ To facilitate the data collection process and streamline statistical analysis, we assigned numbers ranging from 1 to 17 to the vertebrae from T1 to L5 (e.g., T12 was assigned the number 12, and L1 was assigned the number 13). That is to say, the smaller the vertebral number, the higher its location. The apex of LL (LA) was defined as the most anterior lumbar vertebra or intervertebral disc from the C2 plumb line. We assigned numbers ranging from 1 to 5 to the vertebrae from L1 to L5; therefore, when the apex was located at the disc between L3 and L4, the LA value was recorded as 3.5.

To establish an equivalent control group, we assembled a cohort of individuals who did not exhibit TOLF by referencing a database of patients who had undergone whole-spine lateral radiographs and sagittal MRI due to concurrent complaints of lower back discomfort within the same study timeframe. In order to mitigate the impact of confounding variables associated with variances in patient demographics, we employed propensity score matching. In addition to aligning physical attributes, we also harmonized PI, SVA, and TPA to account for disparities in spinal configuration attributed to PI and sagittal misalignment stemming from degenerative alterations. Each TOLF patient was paired with a counterpart from the control group using the nearest neighbor technique, aligning age, gender, and BMI. The matching ratio between the TOLF group and the control group was set at 1:2, and individuals with compression fractures or spondylolisthesis of grade 2 or higher, which could potentially affect spinal shape, were excluded from recruitment into the control group. We ensured that the pooled standard deviations of estimated logits remained below 0.2.

**Figure 1.**
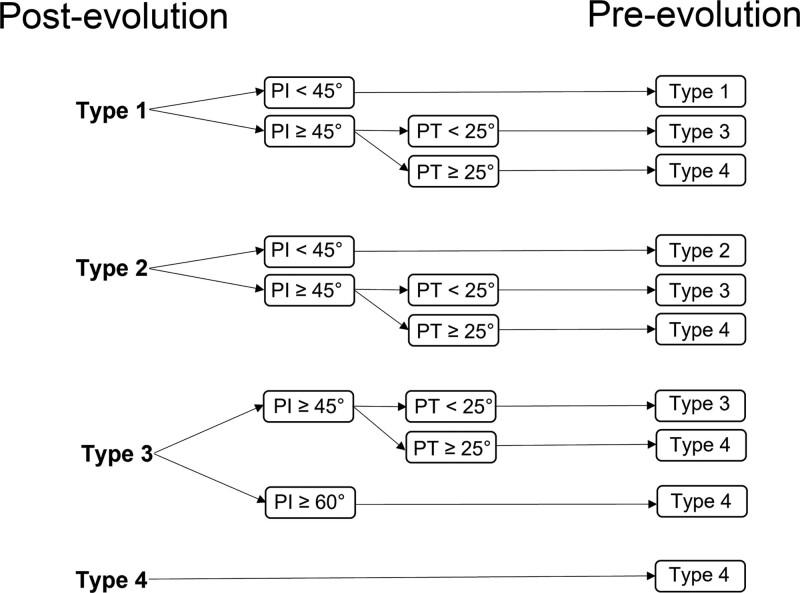
Determination of original, pre-evolutionary spine shape in patients with thoracic ossification of the ligamentum flavum. (A) Post-evolutionary spine type is assigned using the classical Roussouly classification. (B) Pre-evolutionary type is then estimated based on individual PI and PT values. PI = pelvic incidence, PT = pelvic tilt.

Two sets of the pre-and post-evolution Roussouly classification were created to describe the pathologic change of the possible spinal shape in the development of TOLF. First, patients with TOLF may present as post-evolution Roussouly type, indicating a “false” spine type. To determine the pre-evolution Roussouly type, i.e., the original sagittal alignment, the patients were retrospectively classified using their individual PI and PT values based on the modified algorithm (shown in Fig. 1) from Sebaaly et al.^[15]^

Two independent spine fellowship-trained surgeons reviewed all image data. Radiographic parameters were assessed using validated software (Surgimap; Nemaris, Inc., New York, NY). Duplicate measurements were performed, and the average of these 2 measurements was used. The consensus among them determined assessments of IP, LA, and the categorization.

Continuous variables were presented as the mean and standard deviation, while categorical data were presented as the percentage of the number of patients. Subsequent to the propensity score-based matching process, we conducted comparisons of demographic characteristics and radiographic measurements between the 2 cohorts. The Pearson chi-squared test was conducted to compare the distribution of different types of sagittal profiles before and after evolution. The significance was accepted for a *P* value less than .05.

## 3. Results

This study encompassed a total of 43 participants (comprising 22 females and 21 males) with an average age of 64.21 ± 11.01 years (ranging from 43–81), all of whom had undergone posterior decompression surgery for TOLF. In terms of the operated levels, the majority of patients (48.8%) underwent surgery at the T10-11 level, followed by T11-12 (27.9%) and T9-10 (18.6%) within the lower thoracic region. In a smaller proportion, 1 patient each (2.3%) underwent surgery at the T3-4 and T4-5 levels, respectively. A summary of the demographic and radiological data for both groups can be found in Table 1. Radiological assessments revealed that the TOLF group had a significantly lower PT (17.69 ± 7.89) than the control group (21.05 ± 6.11, *P* = .033), while their LL and SS measurements were statistically similar (38.46 ± 11.76 vs 40.08 ± 11.14, *P* = .520) when the 2 groups had a statistically matched PI (50.81 ± 9.56 vs 53.42 ± 7.25, *P* = .163, respectively). Furthermore, the TOLF group demonstrated a statistically higher LDI value (80.20 ± 27.71) than the control group (69.86 ± 20.04, *P* = .046), and the mean value of IP in the TOLF group, measuring 13.92 ± 1.10, exceeded that of the control group (12.95 ± 1.43, *P* = .001), implying a more caudal positioning of IP in the TOLF group.

**Table 1:** Demographics and radiological parameters of the patients with thoracic ossification of ligamentum flavum (TOLF) and control group.

		TOLF group (n = 43)	Control group (n = 86)	*P* value
Demographics	Age (yr)	64.21 ± 11.01	65.02 ± 8.09	.701
	Gender (F/M)	22/21	44/42	1.000
	BMI (kg/m^2^)	26.48 ± 3.66	25.25 ± 4.25	.754
Radiological parameters	SVA (mm)	18.06 ± 30.02	15.88 ± 20.24	.619
	TPA (°)	13.59 ± 8.50	15.01 ± 7.65	.811
	TK (°)	−29.26 ± 9.07	−26.91 ± 11.92	.416
	PI (°)	50.81 ± 9.56	53.42 ± 7.25	.163
	SS (°)	33.11 ± 8.61	32.37 ± 7.43	.675
	PT (°)	17.69 ± 7.89	21.05 ± 6.11	**.033**
	LL (°)	38.46 ± 11.76	40.08 ± 11.14	.520
	LLL (°)	29.41 ± 9.11	27.46 ± 8.74	.319
	PI-LL (°)	12.35 ± 11.25	13.34 ± 9.32	.661
	LDI (%)	80.20 ± 27.71	69.86 ± 20.04	**.046**
	IP	13.92 ± 1.10	12.95 ± 1.43	**.001**
	LA	4.22 ± 0.61	3.84 ± 0.77	.053

Bold font indicates statistically significant.

BMI = body mass index, IP = inflection point, LA = lumbar apex, LL = lumbar lordosis, LLL = lower lumbar lordosis, PI = pelvic incidence, PT = pelvic tilt, SS = sacral slope, SVA = sagittal vertical axis, TK = thoracic kyphosis, TPA = thoracic pelvic angle.

The distribution of patients showed a statistical difference (*P* = .000) between the post- and pre-evolution Roussouly classification (Table 2). According to the post-evolution Rossouly classification, type 2 shape was the most frequently observed type (n = 23, 53.5%). Using the pre-evolution Roussouly classification, 51.5% of the patients (n = 22) were retrospectively classified as type 3, which was the most common type observed.

**Table 2:** Composition of the patients with thoracic ossification of ligamentum flavum according to the post-and pre-evolution Roussouly classification.

	Post-evolution (n, %)	Pre-evolution (n, %)	*P* value
Type 1	6 (14.0)	5 (11.6)	**.000**
Type 2	23 (53.5)	9 (20.9)	
Type 3	9 (20.9)	22 (51.1)	
Type 4	5 (11.6)	7 (16.3)	

Bold font indicates statistically significant.

**Figure 2.**
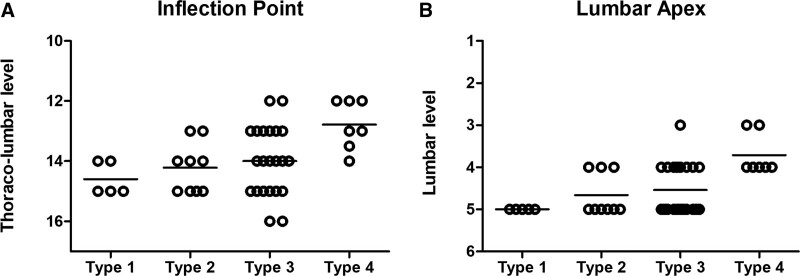
Distribution of the IP and LA values. The data plots show the distribution of the IP (A) and LA (B) values of the patients with TOLF. Horizontal solid lines indicate the median values. IP = inflection point, LA = lumbar apex.

Table 3 presents the average values of the morphologic index in the pre-evolution Roussouly classification, and Figure 2 illustrates the distribution of individual IP and LA. The IP and LA of type 3 showed a similar distribution to type 2, and the mean value of LDI (77.98 ± 8.08) was increased to a level close to that of the standard normal value of type 2 (80%).^[16,17]^

**Table 3:** Morphological index of the patients with thoracic ossification of ligamentum flavum.

	Type 1	Type 2	Type 3	Type 4
PI (°)	37.40 ± 6.18	42.55 ± 3.50	52.54 ± 3.99	65.57 ± 4.99
SS (°)	24.40 ± 6.76	28.22 ± 7.20	35.09 ± 7.92	39.42 ± 6.34
PT (°)	13.00 ± 1.58	14.33 ± 4.24	17.45 ± 8.11	26.14 ± 7.81
LL (°)	30.20 ± 6.37	34.22 ± 11.77	39.22 ± 11.48	47.42 ± 10.70
LLL (°)	32.00 ± 3.24	26.66 ± 8.73	29.27 ± 10.05	31.57 ± 9.82
LDI (%)	108.00 ± 12.72	78.73 ± 6.41	77.98 ± 8.08	69.20 ± 9.35
IP	14.62 ± 0.54	14.22 ± 0.83	13.95 ± 1.10	12.79 ± 0.80
LA	4.90 ± 0.27	4.82 ± 0.66	4.68 ± 0.48	3.83 ± 0.34

IP = inflection point, LA = lumbar apex, LDI = lordosis distribution index, LL = lumbar lordosis, LLL = lower lumbar lordosis, PI = pelvic incidence, PT = pelvic tilt, SS = sacral slope.

## 4. Illustrative case

**Figure 3.**
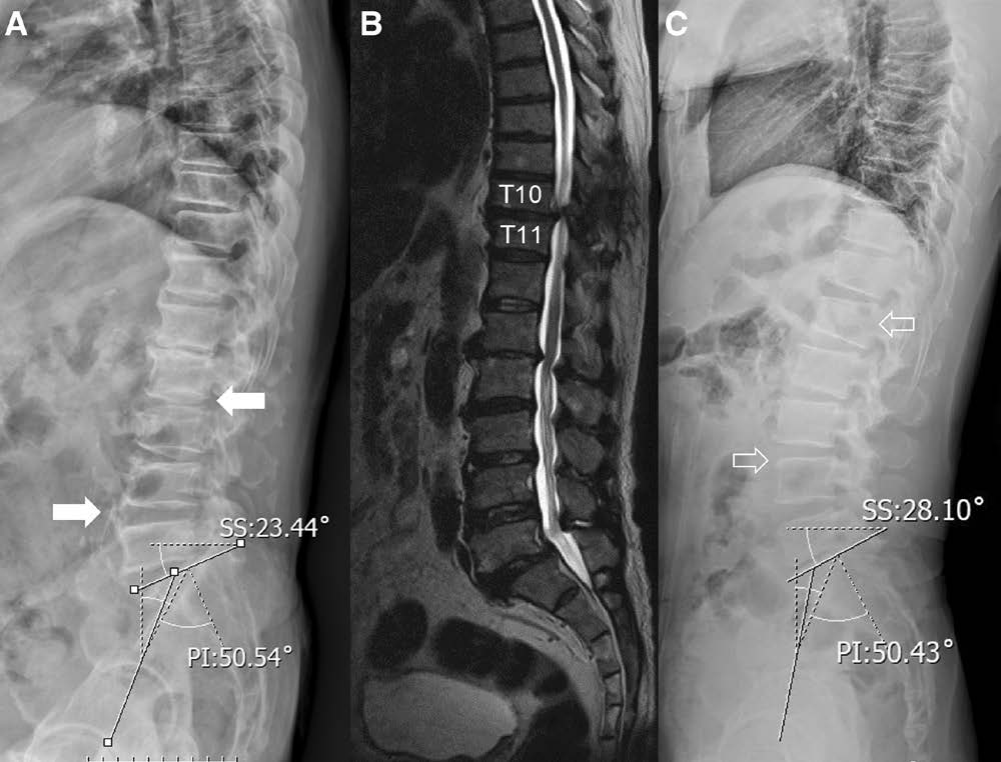
(A) The whole spine lateral radiograph shows a patient with a false Roussouly type 2, characterized by a SS of 23.44° and evolved from Roussouly type 3 with a PI of 50.54°. Left-sided white arrow indicates the IP at the upper endplate of L2 and the right-sided white arrow indicates the LA at L4-5 disc space. (B) The T2-weighted sagittal MR image reveals compression of the thoracic spinal cord at the T10-11 level due to TOLF. (C) A patient in the control group with a PI of 50.43° in Roussouly type 3. The left-sided empty arrow points to the IP located at L1, and the right-sided empty arrow indicates the LA at the upper endplate of L4. IP = inflection point, LA = lumbar apex, PI = pelvic incidence, SS = sacral slope, TOLF = thoracic ossification of the ligamentum flavum.

A male patient, aged 62, is presenting with paraparesis and paresthesia in both lower limbs as a result of thoracic spinal cord compression at the T10-11 level caused by ossification of the ligamentum flavum in the thoracic region (Fig. 3). The patient exhibits a false Roussouly type 2 spinal configuration, as evident from the SS of 23.44°. This particular spinal configuration potentially arises as a pathological progression from a Roussouly type 3, as indicated by the PI of 50.54°. Notably, a downward shift in the IP is observed at the L2-3 level (left-sided arrow), deviating from the expected location at the L1 level for type 3. Furthermore, the LA is positioned at L4-5 (right-sided arrow) rather than the typical L4 placement. The LL is 28.24°, while the lower arc lordosis measures 22.37°. The LDI, which reflects the extent of lordosis distribution along the spine, is found to be 79.2%, indicating an elevated value compared to the expected 70% for type 3. This suggests an elongated and relatively flat lumbar lordosis, characteristic of type 2.

## 5. Discussion

Many prior studies^[17–19]^ have drawn attention to understanding the spinal sagittal balance is of prime importance for treating various spinal pathologies. Roussouly et al^[12]^ introduced a classification for the sagittal shape of the asymptomatic population depending on the SS orientation. They shed light on a geometrical relation between SS and the lower arc of LL (between the horizontal line through the LA and the S1 plateau), and 4 distinct types were proposed accordingly: type 1 and 2 for low SS (<35°), type 3 for average SS (35°< SS < 45°), and type 4 for high SS (>45°). However, the fundamental drawback of this classification is the adoption of a positional parameter, SS, rather than a morphological one. SS, representing the tilted status of the upper endplate of S1 at a particular pelvic position, could be smaller than the original due to pelvic compensatory retroversion. It means patients with degenerative or pathologic status may present themselves as a false spine type. e.g., a patient with sagittal imbalance with reduced SS resulting in mainly Type 1 or 2, despite a large PI.^[15]^ Besides, this depiction was based on an asymptomatic young population and was criticized for impracticality in degenerative or pathologic conditions. To address this issue, Sebaaly et al^[15]^ proposed an algorithm that illustrates how the original types are transformed into their pathological shapes. They theorized that when degenerative changes in the lumbar spine affect a type 3 spine, compensation can occur that results in retroversion with a flat spine and a decrease in SS, resembling a false type 2. Similarly, a reduction in lordosis in a type 4 spine can lead to a type 3 shape with increased PT, creating a false type 3 spine. Practically, false type 2 and type 3 had equal PI, while false type 3 and type 4 are marked by substantially high PIs.

In the present study, the average value of SS in patients with TOLF was 33.11 ± 8.61°, less than 35° as in a typical type 2 spine, while the average value of PI was 50.81 ± 9.56, as in a type 3 spine. Additionally, the analysis by pre-evolution Roussouly classification showed that the majority of patients (51.1%, n = 22) were classified as type 3. Based on these findings, the authors used a backward estimation with the current PI and PT and concluded that the higher proportion of type 2 in patients with TOLF may be a pathological evolution from the original type 3 spine. In addition, while the control group presented radiological characteristics consistent with Roussouly type 3, such as 70% of LDI values and the IP located at L1, the TOLF group displayed features more akin to Roussouly type 2. These observations lend further credence to the authors’ proposed theory. It is worth noting that in addition to this, patients with TOLF were not experiencing sagittal imbalance, as indicated by the fact that their global sagittal parameters of SVA (18.06 ± 30.02 mm) or TPA (13.59 ± 8.50°) were within a balanced range, and their retroverted pelvis was unremarkable (PT = 17.69 ± 7.89°) with a favorable PI-LL mismatch (12.35 ± 11.25°). The current findings also support the hypothesis that the false type 2 shape of TOLF patients may pathologically evolve from the original type 3, which has a high PI and superior compensation capacity.

The pathologic evolution to false type 2 shape may provide a biomechanical clue to understanding the development of TOLF. Roussouly type 2 spine is characterized by low PI (<45°) and related low SS (<35°). The LA is located in the lower lumbar area, yielding a long and flat lordosis resembling a straight line. It is a harmonious flat back with a horizontally oriented disc, resulting in maximal disc pressure; therefore, a person with such a flat back may be at risk for discopathies in the thoracolumbar transitional zone and facet arthritis.^[20,21]^ Recently, Chang et al^[10]^ conducted a cross-sectional case-control study and found that the proportion of patients with classical type 2 alignment was relatively higher in the TOLF group (n = 20, 46.5%) than in the control group (n = 31, 36.0%). They also reported that the TOLF group had a significantly smaller SS and LL, and a higher PI-LL value than the control group. The interpretation of the results was as follows: the reciprocal decrease in TK from PI-LL mismatch is a form of compensatory action causing excessive movements in the thoracolumbar junction. In patients with type 2, greater movements in the thoracolumbar junction might be required due to an insufficient TK angle and lack of compensatory ability by nature. It would end up with an increased mechanical burden enough to develop TOLF. However, it is important to consider that this description was based on patients with a mean age of 69.5 (range 41–86) years and that degenerative changes in the spinopelvic configuration (such as a decrease in SS) may have occurred. As a result, caution should be exercised when interpreting the SS-based Roussouly classification.

In an average individual with a decent sagittal balance, 2 successive curves, including a thoracic kyphotic and lumbar lordotic portion, collaborate to maintain an erect posture with minimal muscular effort.^[22]^ Roussouly et al employed IP as a crucial parameter in analyzing the average spinal figures of a spinopelvic composition.^[23]^ They advocated that the level of the IP was a critical parameter that determined the number of the length of the kyphotic or lordotic vertebrae and the demarcation at which the direction of the arc shifts. The IP was reported to be linked with the TK, and the predictive formula was TK = 2.190 × IP + 8.809 (*R*^2^ = 0.194),^[14]^ implying that as the level of IP moves caudally, the magnitude of TK should be amplified proportionally, and vice versa. Within the normal population exhibiting Roussouly type 3 characteristics, characterized by PI values ranging from 45° to 60°, it has been established that the IP is typically situated at the L1 level.^[16,17]^ In our present investigation, when examining a control group with a PI measuring 53.42° ± 7.25°, the IP is consistently observed at a value of 12.95 ± 1.43, signifying its close association with the L1 level. However, in individuals diagnosed with TOLF, despite having a similar PI (50.81 ± 9.56, *P* = .163) the IP registers at 13.92 ± 1.10, positioning it at around the L2 level. This observation suggests a descent in the IP level, which in turn leads to an elongation of the TK.

The extended TK of the patients with TOLF supports the existing evidence that increased tensile stress on the thoracic ligamentum flavum may facilitate the ossification process.^[24–27]^ At the lower thoracic spine with kyphotic alignment, the ligamentum flavum is persistently subjected to distraction force along its longitudinal axis due to rotational flexion movement located far from the center of the movement.^[24]^ Tsukamoto et al^[25]^ loaded repetitive tensile stress to the rat caudal vertebrae in vivo, and observed ectopic cartilaginous formations surrounded by proliferating round cells near the insertion of the spinal ligaments. The hypothesis was also solidated by Yayama et al,^[5]^ who found a marked coarse arrangement of fragmented and hypertrophied elastic fibers in the non-ossified area of the ligamentum flavum near its origin and attachment. After observing many additional chondrocytes or chondroblast-like cells associated with ligament degeneration, they concluded that the mechanical tensile stress might result in the transformation of elastic fibers into ossified tissue, probably involving undifferentiated mesenchymal cells. Many authors^[28–31]^ have observed that most cases of TOLF occur at the lower thoracic segment, which is a transitional area vulnerable to mechanical stress. The increased tensile strength from the descent of the IP may be a plausible explanation for this.

The causes of TOLF’s pathogenesis are thought to be multifaceted. The high prevalence of TOLF in the East Asian population has led some researchers to investigate the role of genetics^[6,7,29,31]^ and metabolic factors, including DM or truncal obesity, has been presumed to be the cause of TOLF.^[32,33]^ The subjects of the current study are East Asian having high BMI (26.48 ± 3.66 kg/m^2^). Accordingly, the authors acknowledge that the change of spinal shape with the resultant increased tensile force is not the sole explanation for the development of TOLF. However, various studies^[10,28–31]^ have identified mechanical stress as a critical element in TOLF formation. Therefore, we believe this study is valuable because it examined the mechanical pathogenesis of TOLF formation and the role of sagittal spinal shape evolution.

Although this study provides some evidence, there are several limitations to consider. Firstly, the sample size for the study was relatively small due to the rarity of the condition, which may have influenced the statistical results. Secondly, the study’s descriptive and observational design raises the question of whether the evolution from Roussouly type 3 to false type 2 can definitively distinguish between TOLF and general degenerative changes in the spine. Additionally, it is unclear whether the specific spinal shape is the result or cause of TOLF, as the order of incidence was not established in the study. Furthermore, while various factors are believed to contribute to the development of TOLF, the evolution profile of remaining type 1 or type 4 is still uncertain and has not been fully explained.

## 6. Conclusions

The present study analyzed the sagittal spinal shape of TOLF patients. The authors found that the majority of the patients had a false type 2 spine, which had evolved pathologically from Roussouly type 3 and exhibited increased LDI and a lowered level of IP. These changes of spinal shape, including the transition to long hypolordosis and increased length of the TK, may have accentuated tensile stress at the lower thoracic spine and contributed to the development of TOLF.

## Author contributions

**Conceptualization:** Jong-Tae Kim.

**Data curation:** Yong-Hyuk Choi.

**Formal analysis:** Yong-Hyuk Choi.

**Investigation:** Myung Hoon Shin.

**Supervision:** Jong-Tae Kim.

**Visualization:** Myung Hoon Shin.

**Writing – original draft:** Yong-Hyuk Choi, Myung Hoon Shin.
